# Cundurango

**Published:** 1871-09

**Authors:** Alex. Y. P. Garnett


### Cundurango.

During the past six weeks I have been in almost daily receipt of communications from medical gentlemen in different sections of the country, requesting some reliable information relative to the specific and marvellous virtues claimed for the cundurango, as a remedy in the treatment of cancer.

As it would involve the expenditure of more time than I can spare to reply to these several letters of inquiry separately, I have determined to avail myself of your courtesy to make public such information as I have been able to obtain, and the conclusions to which I have arrived, touching the merits of this new so-called remedy for the cure of cancer.

The source from which the drug is obtained, the mode of preparation and administration, together with the romantic and apochryphal history of its discovery, have already been published to the world. It is not necessary, therefore, for me to refer to such publication further than to observe that the question very naturally suggests itself to the medical mind, what proof have we that this individual was affected with cancer at all? Does the diagnostic evidence of an ignorant, non-professional woman furnish sufficient scientific testimony to enable us to believe that this was really a case of that disease? Do not all the circumstances connected with this story justify the opinion that it is a sensational fiction invented for the occasion, or a traditional superstition of the ignorant inhabitants?

Passing from this question of its discovery, we take the drug as presented to us in this country. It seems that specimens of the cundurango were distributed sometime during the month of April last by the Department of State to the Surgeon General of the United States Army, to the Chief of the Bureau of Medicine and

Surgery of the United States Navy, and to the Department of Agriculture, in order that the remedy should be subjected to the test of chemical analysis, as well as therapeutic experimentation by scientific and competent medical men. In accordance with this purpose a chemical analysis of the cundurango was made by Professor Antisell, of this city, an accomplished and experienced practical chemist, the result of which is here given, as an extract from his report:

"Fatty matter, soluble in ether and partially in strong alcohol, .7; yellow rosin, soluble in alcohol, 2.7; starch, gum, and glucose, .5; tannin, yellow and brown coloring matter and extractive, 12.6; cellulose liquin, etc., 64.5.

"On distillation, no volatile oil or acid was obtainable; no crystalline, alkaloid, or active principle was separable by the usual method of proximate analysis. Whatever medical virtues the plant may possess must reside either in the yellow rosin or in the extractive. The former is soluble in alcohol, the latter in water. In the water decoction some of the rosin is diffused, but the greater portion of the rosin is not extracted by water. The therapeutic position of the plant, judged from analysis, is among the aromatic bitters.

It will thus be seen that, unlike almost all of those vegetable growths which possess very powerful and radical therapeutic influences upon the animal economy—such as nux vomica, cinchona, opium, stramonium, etc., etc.—it contains no active principle capable of being eliminated by the ordinary modes of chemical analysis, and hence it is fair to suppose that it really possesses none, and that its virtues, if it possesses any, are dependent chiefly, if not entirely, upon the resinous principle which is insoluble in water; and yet we are told that it was the decoction or infusion of the drug which was used by the humane and affectionate wife, in her first efforts to dispose of her husband, which first developed its wonderful virtues.

Through the courtesy of Surgeon Norris, of the United States Army, I have been permitted to read his report, made to Surgeon General Barnes, detailing the results of his experience with the cundurango in the treatment of cancer, and, although not authorized to do so by publication, I shall take the liberty of stating that, although the case selected for experiment by him presented all the conditions required for a fair test of its merits, it utterly failed to arrest the progress of the disease, or in any decided manner to modify its character or mitigate the suffering of the patient. The case terminated fatally at the expiration of five weeks from the commencement of this treatment, notwithstanding its uninterruped administration during that period. I will here add, that exactly similar results followed in another case which was treated in New York with the cundurango, under instructions from the Surgeon General. In both of these cases, specimens of the morbid products procured after death were submitted to microscopical and other tests to confirm the accuracy of diagnosis.

I am also indebted to the gentlemanly assistant, to the chief of the Bureau of Medicine and Surgery, United States Navy, for the privilege of perusing a lengthy communication upon the subject of cundurango, made to that bureau by the accomplished pharmacist, Dr. E. R. Squibb, of Brooklyn, in which he very clearly exhibits his entire want of confidence in its merits as a cure for cancer, and classifies it with the numerous empirical agents which have, from time to time, heretofore agitated the public mind and disappointed the hopes and expectations of so many unfortunate victims of this terrible malady. I omitted to mention that two other cases of cancer were treated with the cundurango by a medical officer of the army in this city, but with no favorable results.

In view of these facts, together with others equally impressive which might be stated, I am irresistibly forced to the conclusion that the cundurango possesses no value whatever as a remedial agent in the treatment of cancer; that it is capable, indeed, of doing indirect injury by disturbing the functions of the stomach and impairing nutrition; that, so far as I have been able to learn, not a single well-authenticated case of cancer has been cured by its use; that I will venture to affirm there is not a physician whose integrity and veracity can be relied upon, here or elsewhere, who will declare that he has cured a case of cancer by the use of the cundurango, and that he is prepared to prove it by exhibiting his patient to the test of competent medical judges.

I am aware that some importance has been given to the testimony of Vice-President Colfax in favor of a cure having been effected by the use of this remedy upon a member of his family, but does it occur to the public that the honorable gentleman and his friends may have been mistaken in the diagnosis? Was this a case of genuine cancer? and is the disease permanently cured, if so? In a medical and diagnostic point of view, the testimony of Mr. Colfax is, of course, entitled to no more weight than that of any other layman.

In conclusion, I would simply remark that the apparent and conceded attempt to manufacture a fictitious reputation for this drug, resting as it does upon such questionable data, seems rather to have been inspired by the dazzling prospect of pecuniary speculation, than by a laudable desire to advance the triumphs of science, or to alleviate the sufferings of unfortunate humanity.—ALEX. Y. P. GARNETT, in *Jour. of Phar.*

